# From SLAM to Situational Awareness: Challenges and Survey

**DOI:** 10.3390/s23104849

**Published:** 2023-05-17

**Authors:** Hriday Bavle, Jose Luis Sanchez-Lopez, Claudio Cimarelli, Ali Tourani, Holger Voos

**Affiliations:** 1 Interdisciplinary Center for Security Reliability and Trust (SnT), University of Luxembourg, 1855 Luxembourg, Luxembourg; joseluis.sanchezlopez@uni.lu (J.L.S.-L.); claudio.cimarelli@uni.lu (C.C.); ali.tourani@uni.lu (A.T.); holger.voos@uni.lu (H.V.); 2 Department of Engineering, Faculty of Science, Technology, and Medicine (FSTM), University of Luxembourg, 1359 Luxembourg, Luxembourg

**Keywords:** simultaneous localization and mapping, scene understanding, scene graphs, mobile robots

## Abstract

The capability of a mobile robot to efficiently and safely perform complex missions is limited by its knowledge of the environment, namely the *situation*. Advanced reasoning, decision-making, and execution skills enable an intelligent agent to act autonomously in unknown environments. Situational Awareness (SA) is a fundamental capability of humans that has been deeply studied in various fields, such as psychology, military, aerospace, and education. Nevertheless, it has yet to be considered in robotics, which has focused on single compartmentalized concepts such as sensing, spatial perception, sensor fusion, state estimation, and Simultaneous Localization and Mapping (SLAM). Hence, the present research aims to connect the broad multidisciplinary existing knowledge to pave the way for a complete SA system for mobile robotics that we deem paramount for autonomy. To this aim, we define the principal components to structure a robotic SA and their area of competence. Accordingly, this paper investigates each aspect of SA, surveying the state-of-the-art robotics algorithms that cover them, and discusses their current limitations. Remarkably, essential aspects of SA are still immature since the current algorithmic development restricts their performance to only specific environments. Nevertheless, Artificial Intelligence (AI), particularly Deep Learning (DL), has brought new methods to bridge the gap that maintains these fields apart from the deployment to real-world scenarios. Furthermore, an opportunity has been discovered to interconnect the vastly fragmented space of robotic comprehension algorithms through the mechanism of *Situational Graph (S-Graph)*, a generalization of the well-known scene graph. Therefore, we finally shape our vision for the future of robotic situational awareness by discussing interesting recent research directions.

## 1. Introduction

The robotics industry is experiencing an exponential growth, embarking on newer technological advancements and applications. Mobile robots have gained interest from a commercial perspective due to their capabilities to replace or aid humans in repetitive or dangerous tasks [1]. Already, many industrial and civil-related applications employ mobile robots [2]. For example, industrial machines and underground mines’ inspections, surveillance and road-traffic monitoring, civil engineering, agriculture, healthcare, search, and rescue interventions in extreme environments, e.g., natural disasters, for exploration and logistics [3].

On one hand, mobile robots can be controlled in manual teleoperation or semi-autonomous mode with constant human intervention in the loop. Furthermore, teleoperated mobile robots can be apprehended using applications such as augmented reality (AR) [4], ref. [5] to enhance human–robot interactions. On the other hand, in fully autonomous mode, a robot performs an entire mission based on its understanding of the environment given only a few commands [6]. Remarkably, autonomy reduces the costs and risks while increasing productivity and is the goal of current research to solve the main challenges that it raises [7]. Unlike the industrial scenario, where autonomous agents can act in a controlled environment, mobile robots operate in the dynamic, unstructured, and cluttered world domain with little or zero previous knowledge of the scene structure.

**Figure 1:**
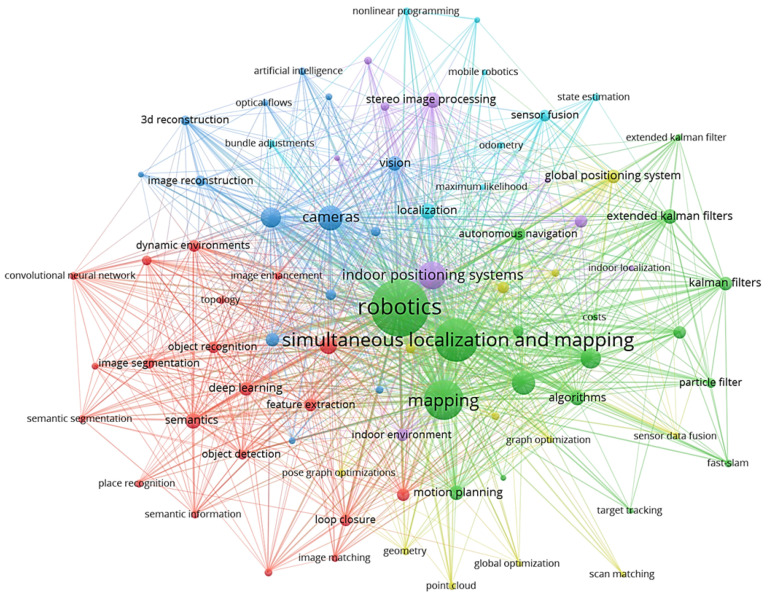
Scopus database since 2015 covering the research in *Robotics* and *SLAM*. All the works focused on independent research areas which could be efficiently encompassed in one field of Situational Awareness for robots.

Up to now, the robotics community has focused chiefly on research areas such as sensing, perception, sensor fusion, data analysis, state estimation, localization and mapping, i.e., Simultaneous Localization and Mapping (SLAM), and Artificial Intelligence (AI) applied to various image processing problems, in a compartmentalized manner. Figure 1 shows the mentioned targets’ data obtained from *Scopus* abstract and citation database. However, autonomous behavior entails understanding the situation encompassing multiple interdisciplinary aspects of robotics, from perception, control, and planning to human–robot interaction. Although SA [8] is a holistic concept widely studied in fields such as psychology, the military, and in aerospace, it has been barely considered in robotics. Notably, Endsley [9] formally defined SA in the 1990s as “*the perception of the elements in the environment within a volume of time and space, the comprehension of their meaning and the projection of their status shortly*”, which remains valid to date [10]. Hence, we turn this definition into the perspective of mobile robotics to derive a unified field of research that garners all the aspects required by an autonomous system.

**Figure 2:**
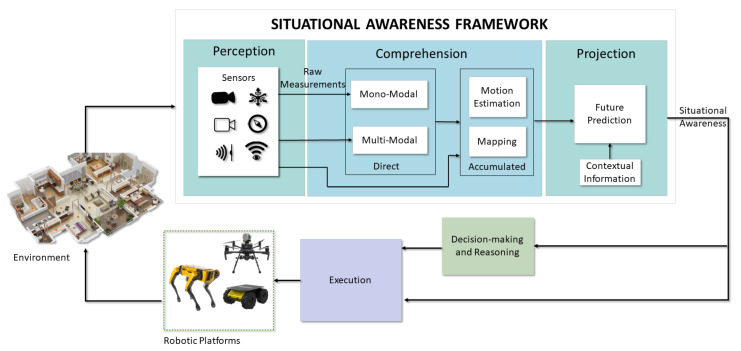
The proposed Situational Awareness system architecture for autonomous mobile robots. We break it into its principal components, namely perception, comprehension, and projection, and show how they are connected.

Therefore, a robot’s Situational Awareness (SA) system must continuously acquire new observations of the surroundings, understand its essential elements and make complex reasoning, and project the world state into a possible future outcome to make decisions and execute actions that would let it accomplish its goals. Accordingly, we depict in Figure 2 a general architecture of SA that divides the specific competence areas into three layers, with an increasing level of intelligence. Thus, we raise the following research question:

- *What are the components of a robot’s situational awareness system?*

To answer the mentioned question, we characterize SA by its three main parts, for which we propose a description that delimits their scope and defines their purpose:

The **perception of the situation** consists of the acquisition of exteroceptive information, i.e., of the surroundings, such as visual light intensity or distance, and proprioceptive information, i.e., of the internal values of the robot, such as velocity or temperature, and information on the situation. Sensors provide raw measurements that must be transformed to acquire actual knowledge or may directly inform the robot about its state with little processing. For instance, active range sensors provide a distance to objects by well-defined models. On the contrary, the pixel intensity values of a camera, other than being distorted by unknown parameters, depend on complex algorithms to extract meaningful depth, which is still ongoing research. Considering this, multiple sensor modalities are essential to perceive complementary details of the situation, e.g., the acceleration of the robot and the metric scale, the visible light intensity and its changes, and compensate for low performance in different conditions, e.g., low light, light-transparent materials, or fast motion. Hence, perception includes the array of sensors that give each robot specific attributes and those algorithms that augment the amount of information at the disposal of successive layers. Furthermore, as cameras are the predominant source of mostly latent environmental features, image processing algorithms are necessary to gain insights into the situation from these sensors. We include such basic algorithms, which usually require a single image frame, into the next layer of direct comprehension.

The **comprehension of the situation** extends from understanding the current perception, considering the possible semantic relationships, to build a short-term understanding using perceptual observation at a given time instant, referred to as *direct situational comprehension*, or a long-term model that includes the knowledge acquired in the past, namely, the *accumulated situational comprehension*. Multiple abstract relationships can be created to link concepts in a structured model of the situation, such as geometric (e.g., the shape of the objects), semantic (e.g., the type and functionality of the objects), topological (e.g., the order in the space), ontological (e.g., the hierarchy of commonsense concepts), dynamic (e.g., the motion between the objects), or stochastic (e.g., to include uncertainty information). In addition, the comprehension of the situation is affected by mechanisms such as attention that are controlled by the decision-making and control processes (e.g., looking for a particular object in a room vs. getting a global overview of a room).

The **projection of the situation** into the future is essential for decision-making processes, and a higher level of comprehension facilitates this ability. A more profound understanding of the environmental context, which includes information such as the robot’s position, velocity, pose, and any static or dynamic obstacles in the surrounding area, can lead to a more accurate projection model. The projection process involves forecasting the future states of both the ego-agent and external agents to predict behaviors and interactions, enabling the robot to adapt its actions to achieve its goals effectively.

The rest of this paper aims to delve into those research questions naturally developed as a consequence of the exposed SA topic:

- *What has been achieved so far, and what challenges remain?*
- *What could the future direction of Situational Awareness be?*

Thus, by reviewing the current state-of-the-art methods for mobile robots that may fall into perception, comprehension, and projection, we aim to study the broad field of Situational Awareness as one and understand the advancement and limitations of its components. Then, we discuss in which direction we envision the research will address the remaining challenges and bridge the gap that divides robots from mature, intelligent autonomous systems.

We summarize the main contributions of this paper as:

- Comprehensive review of the state-of-the-art approaches: we conduct a thorough analysis of the latest research related to enhancing situational awareness for mobile robotic platforms, covering computer vision, deep learning, and SLAM techniques.
- Identification and analysis of the challenges: we classify and discuss the reviewed approaches according to the proposed definition of situational awareness for mobile robots and highlight their current limitations for achieving complete autonomy in mobile robotics.
- Proposals for future research directions: we provide valuable insights and suggestions for future research directions and open problems that need to be addressed to develop efficient and effective situational awareness systems for mobile robotic platforms.

## 2. Situational Perception

Situational perception enables robots to perceive their known state as well as the situation around them using a single or a combination of onboard proprioceptive and exteroceptive sensors. The continuous technological advances regarding chip developments have made many sensors suited for use onboard mobile robots [11], as they come with a small form factor and possibly low power consumption. The primary sensor suite of the average robot can count on a wide array of devices, such as Inertial Measurement Units (IMUs), magnetometers, barometers, and Global Navigation Satellite Systems (GNSS) receivers, e.g., for the typical Global Positioning System (GPS) satellite constellation. Sensors such as IMUs, which can be utilized by robotic platforms to measure their attitude, angular velocities, and linear accelerations, are cheap and lightweight, making them ideal for running onboard the platforms, though the performance of these sensors can degrade over time due to the accumulation of errors coming from white Gaussian noise [12]. Magnetometers are generally integrated within an IMU sensor, measuring the accurate heading of the robotics platform relative to the Earth’s magnetic field. The sensor measurements from a magnetometer can be corrupted when the robot navigates in environments with constant magnetic fields interfering with the Earth’s magnetic field. Barometers can be utilized by flying mobile robots to measure their altitude changes through measured pressure changes, but they suffer from bias and random noise in measurements in indoor environments due to ground/ceiling effects [13]. Wheel encoders are utilized by ground mobile robots to measure the velocities of the platform and obtain its relative position. GNSS receivers, as well as their higher-precision variants, such as Real-Time Kinematic (RTK) or differential GNSS, provide reliable position measurements of robots in a global frame of reference relative to the Earth in outdoor environments. However, these sensors can work reliably in uncluttered outdoor environments with multiple satellites connected or within a direct line of sight with the RTK base station [14].

The adoption of cameras as exteroceptive sensors in robotics has become increasingly prevalent due to their ability to provide a vast range of information in a compact and cost-effective manner [15]. In particular, RGB cameras, including monocular cameras, have been widely used in robotics as primary sensor as it provides the robots with colored images which can be further processed to extract meaningful information from their environment. Additionally, cameras with depth information, such as stereo or RGB-D cameras, have emerged as a dominant sensor type in robotics given that they provide the robot with additional capabilities of perceiving the depth of the different objects within the environment. As such, the use of standard cameras is expected to continue playing a crucial role in developing advanced robotic systems. These standard cameras suffer from the disadvantage of motion blur in the presence of a rapid motion of the robot, and the perceived quality can degrade as the robot navigates in changing lighting conditions.

In robotics, RGB and RGB-D (i.e., with depth) cameras are thus complemented by infrared (IR) cameras, also referred to as thermal cameras when detecting long-wave radiation, for gaining extended visibility during nighttime or adverse weather conditions. These sensors can provide valuable information not detectable by human eyes or traditional cameras, such as heat signatures and thermal patterns. By incorporating thermal and IR cameras into the sensor suite, mobile robots can detect and track animated targets by following heat signatures, and navigating low-visibility environments, thus operating in a broader range of conditions. These specialized sensors can significantly enhance a robotic system’s situational awareness and overall performance.

Neuromorphic vision sensors [16], also known as event cameras [17], such as the Dynamic Vision Sensor (DVS) [18], overcome the limitations of standard cameras by encoding pixel intensity changes rather than an absolute brightness value and providing very high dynamic ranges as well as no motion blur during rapid motions. However, due to the asynchronous nature of event cameras, measurements of the situations are only provided in case of variations in the perceived scene brightness that are often caused by the motion of the sensor itself. Hence, they can measure a sparse set of points, usually in correspondence with edges. To perceive a complete dense representation of the environment, such sensors onboard mobile robots are typically combined with the traditional pixels of RGB cameras, as in the case of the Dynamic and Active-Pixel Vision Sensor (DAVIS) [19] or Asynchronous Time-Based Image Sensor (ATIS) [20] cameras. Nevertheless, algorithms have also been proposed to reconstruct traditional intensity frames by integrating events over time to facilitate the reuse of preexisting image processing approaches [21] or even produce a high-frame-rate video by interpolating new frames [22].

Ranging sensors, such as small-factor solid-state Light Detection and Rangings (LIDARs) or ultrasound sensors, are the second most dominant group of employed exteroceptive sensors onboard mobile robots. One-dimensional LIDARs and ultrasound sensors are used mainly in flying mobile robots to measure their flight altitude but only measure limited information about their environments, while ground mobile robots can utilize the sensor to measure the distance to nearest object. Two- and three-dimensional LIDARs accurately perceive the surroundings in 360°, and the newer technological advancements have reduced their size and weight. However, utilizing these sensors onboard small-sized robotic platforms is still not feasible, and the high acquisition cost hampers the adoption of this sensor by the broad commercial market. Even for autonomous cars, a pure-vision system, which may include event cameras, is often more desirable from an economic perspective.

Frequency-modulated continuous wave (FMCW) radio detection and ranging (RADAR) systems transmit a continuous waveform with a changing frequency over time. This changing frequency creates a frequency sweep, or chirp, continuously transmitted and reflected off objects in the radar’s field of view. An FMCW radar can determine the detected objects’ range, velocity, and angle by measuring the frequency shift between the transmitted and received signals. Millimeter-wave (mmWave) radars use short-wavelength electromagnetic waves in the GHz range to obtain millimeter accuracy and are a valid alternative to LIDAR for range measurements in robotics [23]. Even though they have a lower angular resolution and more limited range than LIDAR, they offer a smaller form factor and a lower cost. Additionally, they can estimate the speed of objects by leveraging the Doppler effect. Nevertheless, mmWave radars can detect transparent surfaces that are challenging to see with LIDAR. As such, they have become an attractive option for robotic applications where cost, form factor, and the detection of transparent surfaces are crucial.

A Radiofrequency (RF) signal is another technology based on signal processing that allows a robot to infer its global position by estimating its distance with one or multiple base stations. Differently from GPS, RF signals may be able to provide positioning information in indoor environments as well, even though range measurements require it to be fused with other sources of motion estimation, e.g., from an IMU. Contrary to mmWave radars, RF-based localization or mapping is far less precise, but newer technology such as 5G promises superior performance. However, a drawback of these approaches is that they require synchronization between the antenna and the receiver for computing a time of arrival (TOA) and possibly line-of-sight (LOS) communication, especially when only one antenna is available. Other RF measurements, such as the time difference of arrival (TDOA), allow the release of the synchronization requirement or the computation of the position in a non-line-of-sight (NLOS) scenario by extracting information from matrices of channel state information (CSI). Kabiri et al. [24] provide an exhaustive review of RF-based localization methods and give an outlook on current challenges and future research directions.

Table 1 summarizes the different sensors used onboard mobile robots with their individual limitations. Given the multifaceted characteristics of the available sensors, relying on a monomodal perception does not guarantee a safe robot deployment in real-world settings. Consequently, multimodal perception is often preferred at the cost of more complex solutions to properly fuse and time-synchronize the measurements from multiple sensors. Nevertheless, it is essential to perceive complementary information of the situation and to build a complete state of the autonomous agent, e.g., the acceleration of a robot, the visual light intensity of the environment, its global position, or the distance with obstacles, and compensate for low performance in different conditions, e.g., dark rooms or low-light, transparent materials, or non-Lambertian surfaces.

The traditional approach to designing robots involves tailoring their sensor selection and configuration to the specific task they are intended to perform. However, this approach may not be sufficient for a humanlike perception capable of adapting to diverse external situations. To overcome this limitation, it is necessary to equip robots with a standard, versatile sensor suite that can provide detailed and accurate information about their surroundings and dynamic elements. This sensor suite, coupled with advanced processing algorithms (explained in Section 3 and Section 4), can enable robots to perceive their environment like humans, irrespective of the application they are designed for.

## 3. Direct Situational Comprehension

Some research works focus on transforming the complex raw measurements provided by sensors into more tractable information with different levels of abstraction, i.e., feature extraction for an accurate scene understanding, without building a complex long-term model of the situation. Table 2 provides a summary of the presented direct comprehension methods with their key limitations while using onboard mobile robots. Direct situational comprehension based on sensor modalities can be divided into two main categories, as described below.

### 3.1. Monomodal

These algorithms utilize a single sensor source to extract useful environmental information. The two primary sensor modalities used in robotics are *vision-based sensors* and *range-based sensors* for the rich and plentiful amount of information in their scene observations.

Vision-based comprehension started with the early works of Viola and Jones [25] presenting an object-based detector for face detection using *Haar-like features* and *Adaboost feature classification*. Following works for visual detection and classification tasks such as [26,27,28,29,30] utilized well-known image features, e.g., Scale-Invariant Feature Transform (SIFT) [31], Speeded-Up Robust Features (SURF) [32], Histogram of Oriented Gradients (HOG) [33], along with Support Vector Machine (SVM)-based classifiers [34]. The mentioned methods focused on extracting only a handful of helpful information from the environment, such as pedestrians, cars, and bicycles, showing degraded performance in difficult lighting conditions and occlusions.

**Figure 3:**
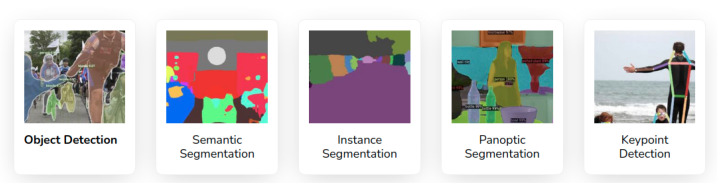
Deep-learning-based computer vision algorithms for monomodal scene understanding. Copyright to [84].

With the establishment of DL in computer vision and image processing for robot vision, recent algorithms in the literature robustly extract the scene information utilizing Convolutional Neural Networks (CNNs) in the presence of different lighting conditions and occlusions. In computer vision, different types of DL-based methods exist based on the kind of scene-extracted information. Algorithms such as *Mask-RCNN* [36], *RetinaNet* [37], *TensorMask* [38], *TridentNet* [39], and *Yolo* [35] perform detection and classification of several object instances, and they either provide a bounding box around the object or perform a pixelwise segmentation for each object instances. Other algorithms such as [40,41,42,43,44] perform a dense semantic segmentation, being able to extract all relevant information from the scene. Additionally, Kirillov et al. [45], Cheng et al. [46] aimed to detect and categorize all object instances in an image, regardless of their size or position, through a panoptic segmentation while still maintaining a semantic understanding of the scene. This task is particularly challenging because it requires integrating pixel-level semantics and instance-level information. Figure 3 showcases different image segmentation algorithms. Two-dimensional *scene graphs* [47,48] could then connect the semantic elements detected by the panoptic segmentation in a knowledge graph that let one reason about relationships. Moreover, this knowledge graph facilitates the inference of single behaviors and interactions among the participants in a scene, animate or inanimate [49].

To overcome the limitations of the visible spectrum in the absence of light, thermal infrared sensors have been researched to augment situational comprehension. For instance, one of the earlier methods [50] found humans in nighttime images by extracting thermal shape descriptors that were then processed by *Adaboost* to identify positive detection. In contrast, newer methods [51,52] utilize deep CNNs on thermal images for identifying different objects in the scene, such as humans, bikes, and cars. Though research in the field of event-based cameras for scene understanding is not yet broad, some works such as [85] present an approach for dynamic object detection and tracking using event streams, whereas [53] present an asynchronous CNN for detecting and classifying objects in real time. *Ev-SegNet* [54] is an approach that introduced one of the first semantic segmentation pipelines based on event-only information.

**Figure 4:**
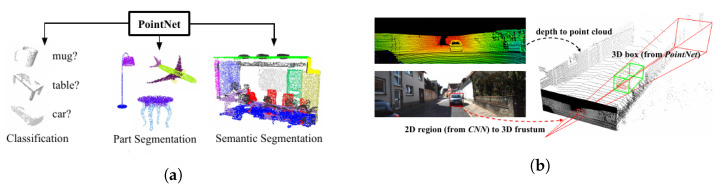
Monomodal and multimodal scene understanding algorithms, (**a**) *PointNet* algorithm using only LIDAR measurements. Copyright to [62]. (**b**) *Frustrum PointNets* algorithm combining RGB and LIDAR measurements improving the accuracy of *PointNet*. Copyright to [79].

Range-based comprehension methods with earlier works such as [55] and ref. [56] presented object detection for range images from 3D LIDAR using an SVM for object classification. However, the authors in [57] utilized range information to identify the terrain around the robot and objects and used an SVMs to classify each category. Nowadays, deep learning is also playing a fundamental role in scene understanding using range information. Some techniques utilize CNNs for analyzing range measurements translated into camera frames by projecting the 3D points onto an abstract image plane. For example, *Rangenet++* [58,59], *SqueezeSeg* [60], and *SqueezeSegv2* [61] project the 3D point-cloud information onto 2D range-based images for performing the scene understanding tasks. The above-mentioned methods argue that CNN-based algorithms can be directly applied to range images without using expensive 3D convolution operators for point cloud data. Others apply CNNs directly on the point cloud information for maximizing the preservation of spatial information. Approaches such as *PointNet* [62] (Figure 4a), *PointNet++* [63], *TangentConvolutions* [64], *DOPS* [65], and *RandLA-Net* [66] perform convolutions directly over the 3D point cloud data to semantically label the point cloud measurements.

### 3.2. Multimodal

The fusion of multiple sensors for situational comprehension allows algorithms to increase their accuracy by observing and characterizing the same environment quantity but with different sensor modalities [86]. Algorithms combining RGB and depth information have been widely researched due to the easy availability of the sensors publishing RBG-D information. González et al. [67] studied and presented the improvement of the fusion of multiple sensor modalities (RGB and depth images), numerous image cues, and various image viewpoints for object detection, whereas Lin et al. [68] combined 2D segmentation and 3D geometry understanding methods to provide contextual information for classifying the categories of the objects and identifying the scene in which they are placed. Several algorithms classifying and estimating the pose of objects using CNNs, such as [69], *PoseCNN* [70], *DenseFusion* [71,72,73], rely extensively on RBG-D information. These methods are primarily employed for object manipulation tasks, using robotic manipulators fixed on static platforms or mobile robots.

Alldieck et al. [87] fused RGB and thermal images from a video stream using contextual information to access the quality of each image stream to combine the information from the two sensors accurately, whereas methods such as *MFNet* [74], *RTFNet* [75], *PST900* [76], and *FuseSeg* [77] combined the potential of RGB images along with thermal images using CNN architectures for the semantic segmentation of outdoor scenes, providing accurate segmentation results even in the presence of degraded lighting conditions. Zhou et al. [88] proposed *ECFFNet* to perform the fusion of RGB and thermal images at the feature level, which provided complementary information, effectively improving object detection in different lighting conditions. Spremolla et al. [89], Mogelmose et al. [90] performed a fusion of RGB, depth, and thermal camera computing descriptors in all three image spaces and fused them in a weighted average manner for efficient human detection.

Dubeau et al. [91] fused the information from an RGB and depth sensor with an event-based camera cascading the output of a deep Neural Network (NN) based on event frames with the output from a deep NN for RBG-D frames for a robust pose tracking of high-speed moving objects. *ISSAFE* [78] is another approach that combines event-based CNN with an RGB-based CNN using an attention mechanism to perform semantic segmentation of a scene, utilizing the event-based information to stabilize the semantic segmentation in the presence of high-speed object motions.

To improve situational comprehension using 3D point cloud data, methods have been presented that combine information extracted over RGB images with their 3D point cloud data to accurately identify and localize the objects in the scene. *Frustrum PointNets* [79] (Figure 4b) performed 2D detection over RGB images which were projected to a 3D viewing frustum from which the corresponding 3D points were obtained, to which a *PointNet* [62] (Figure 4a) was applied for object instance segmentation, and an *amodal* bounding box regression was performed. Methods such as *AVOD* [80,81] extract features from both RGB and 3D point clouds projected to a bird’s eye view and fuse them to provide 3D bounding boxes for several object categories. *MV3D* [82] extract features from RGB images and 3D point cloud data from the front view as well as a bird’s eye view to fuse them in a Region of Interest (RoI) pooling, predicting the bounding boxes as well as the object class. *PointFusion* [83] employs an RGB and 3D point cloud fusion architecture which is unseen and object-specific and can work with multiple sensors providing depth.

*Direct situational comprehension* algorithms only provide the representation of the environment at a given time instant and mostly discard the previous information, not creating a long-term map of the environment. In this regard, the extracted knowledge can thus be transferred to the subsequent layer of *accumulated situational comprehension*.

## 4. Accumulated Situational Comprehension

A greater challenge consists of building a long-term multiabstraction model of the situation, including past information. Even small errors not considered at a particular time instant can cause a high divergence between the state of the robot and the map estimate over time. To simplify the explanation, we divided this section into three subsections, namely, *motion estimation*, *motion estimation and mapping*, and *mapping*.

### 4.1. Motion Estimation

The motion estimation component is responsible for estimating the state of the robot directly, using the sensor measurements from single/multiple sources and the inference provided by the *direct situational comprehension* component (see Section 3.1). While some motion estimation algorithms only use real-time sensor information to estimate the robot’s state, others estimate the robot’s state inside a pregenerated environment map. Early methods estimated the state of the robot based on filtering-based sensor fusion techniques such as an *Extended Kalman Filter (EKF)*, an *Unscented Kalman Filter (UKF)*, and *Monte Carlo Localization (MCL)*. Methods such as those in [92,93] use *MCL*, providing a probabilistic hypothesis of the state of the robot directly, using the range measurements from a range sensor. Anjum et al. [94] performed a *UKF* based fusion of several sensor measurements such as gyroscopes, accelerometers, and wheel encoders to estimate the motion of the robot. Kong et al. [95], Teslic et al. [96] performed an EKF based fusion of odometry from robot wheel encoders and measurements from a prebuilt map of line segments to estimate the robot state, whereas Chen et al. [97] used a prebuilt map of corner features. Ganganath and Leung [98] presented both UKF and MCL approaches for estimating the pose of the robot using wheel odometry measurements and a sparse prebuilt map of visual markers detected with an RGB-D camera. In contrast, Kim and Kim [99] presented a similar approach using ultrasound distance measurements with respect to an ultrasonic transmitter.

The simplified mathematical models are subject to several assumptions that limited earlier motion estimation methods. Newer methods try to improve these limitations by providing mathematical improvements over the earlier methods and accounting for delayed measurements between different sensors, such as the UKF developed by Lynen et al. [100] and the EKF by Sanchez-Lopez et al. [101], which compensate for time-delayed measurements in an iterative nature for a quick convergence to the actual state. Moore and Stouch [102] presented an EKF/UKF algorithm, well-known in the robotics community, which can take an arbitrary number of heterogeneous sensor measurements for the estimation of the robot state. Wan et al. [103] used an improved version of a Kalman filter called the *error-state Kalman filter*, which used measurements from RTK GPS, LIDAR, and IMU for a robust state estimation. Liu et al. [104] presented a *multi-innovation UKF (MI-UKF)*, which utilized a history of innovations in the update stage to improve the accuracy of the state estimate; it fused IMU, encoder, and GPS data and estimated the slip error components of the robot.

The motion estimation of robots using a Moving-Horizon Estimation (MHE) has also been studied in the literature where methods such as in [105] fuse wheel odometry and LIDAR measurements using an MHE scheme to estimate the state of the robot, claiming a robustness over any outliers in the LIDAR measurements. Liu et al. [106], Dubois et al. [107] studied a *multirate MHE* sensor fusion algorithm to account for sensor measurements obtained at different sampling rates. Osman et al. [108] presented a generic MHE-based sensor fusion framework for multiple sensors with different sampling rates, compensating for missed measurement, outlier rejection, and satisfying real-time requirements.

**Figure 5:**
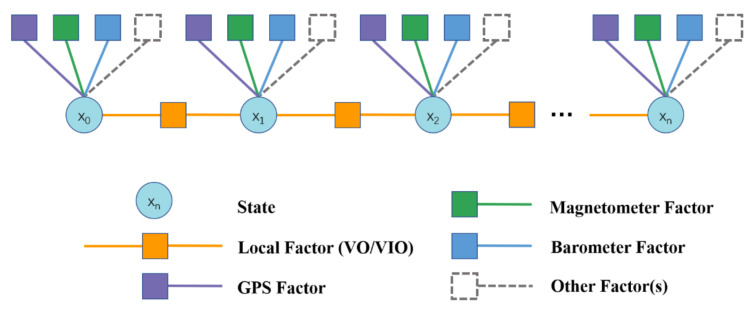
Localization factor graph used for estimating the robot state fusing multiple sensor measurements. Copyright to [116].

Recently, motion estimation algorithms of mobile robots using *factor-graph*-based approaches have also been extensively studied as they have the potential to provide a higher accuracy. Factor graphs can encode either the entire history of the robot state or go back up to a fixed time, i.e., fixed-lag smoothing methods, capable of handling different sensor measurements in terms of nonlinearity and varying frequencies optimally and intuitively (see Figure 5). Ranganathan et al. [109] presented one of the first graph-based approaches using *square-root fixed-lag smoothing* [110], for fusing information from odometry, visual, and GPS sensors, whereas Indelman et al. [111] presented an improved fusion based on an incremental smoothing approach, *iSAM2* [112], fusing IMU, GPS and visual measurements from a stereocamera setup. The methods presented in [113,114] utilized sliding-window factor graphs for estimating the robot’s state by fusing several wheel odometry sources along with global pose sources. Mascaro et al. [115] also presented a sliding-window factor graph combining visual odometry information, IMU, and GPS information to estimate the drift between the local odometry frame with respect to the global frame, instead of directly estimating the robot state. Qin et al. [116] presented a generic factor graph-based framework for fusing several sensors. Each sensor served as a factor connected with the robot’s state, quickly adding them to the optimization problem. Li et al. [117] proposed a novel graph-based framework for sensor fusion that combined data from a stereo visual–inertial navigation system, i.e., S-VINS, and multiple GNSS sources in a semitightly coupled manner. The S-VINS output was an initial input to the position estimate derived from the GNSS system in challenging environments where GNSS data are limited. By integrating these two data sources, the framework improved the robot’s global pose estimation accuracy.

The *motion estimation* algorithms, as illustrated in Figure 5, do not simultaneously create a map of the environment, limiting their environmental knowledge, which has led to research on simultaneous *motion estimation and mapping* algorithms described in the following subsection.

### 4.2. Motion Estimation and Mapping

This section covers the approaches which estimate not only the robot motion given the sensor measurements but also the map of the environment, i.e., they model the scene in which the robot navigates. These approaches are commonly known as SLAM, which is one the widely researched topics in the robotics industry [118], as it enables a robot to use scene modeling without the requirement of prior maps and in applications where initial maps cannot be obtained easily. Vision and LIDAR sensors are the two primary exteroceptive sensors used in SLAM for map modeling [15,119]. As in the case of *motion estimation* methods, SLAM can be performed using a single-sensor modality or using information from different sensor modalities and combining it with scene information extracted from the *direct situational comprehension* module (see Section 3). SLAM algorithms have a subset of algorithms that do not maintain the entire map of the environment and do not perform stages of *loop closure* called *odometry estimation algorithms*, where Visual Odometry (VO) becomes a subset of visual SLAM (VSLAM) and LIDAR odometry a subset of LIDAR SLAM. Table 3 and Table 4 provide a brief summary of the above-presented approaches highlighting the different datasets used in their validation along with their limitations.

#### 4.2.1. Filtering

Earlier SLAM approaches such as in [120,121,122] applied an EKF to estimate the robot pose by simultaneously adding/updating the landmarks observed by the robots. However, these methods were quickly discarded as their computational complexity increased with the number of landmarks, and they did not efficiently handle nonlinear measurements [123]. Accordingly, *FastSLAM 1.0* and *FastSLAM 2.0* [124] were proposed as improvements to EKF-SLAM, which combined particle filters to calculate the trajectory of the robot with individual EKFs for landmark estimation. These techniques also suffered from the limitations of sample degeneracy when sampling the proposal distribution and problems with particle depletion.

#### 4.2.2. Metric Factor Graphs

Modern SLAM, as described in [118], has moved to a more robust and intuitive representation of the state of the robot along with sensor measurements, as well as the environment map to create factor graphs as presented in [110,112,125,126,127]. Factor-graph-based SLAM, based on the type of map used for the environmental representation and optimization, can be divided into *metric* and *metric–semantic* factor graphs.

A metric map encodes the understanding of the scene at a geometric level (e.g., lines, points, and planes), which is utilized by a SLAM algorithm to model the environment. *Parallel tracking and mapping (PTAM)* was one of the first feature-based monocular algorithms which split the tracking of the camera in one thread and the mapping of the key points in another, performing a batch optimization for optimizing both the camera trajectory and the mapped 3D points. Similar extensions to the *PTAM* framework are *ORB-SLAM* [128] and *REMODE* [129] which create a semidense 3D geometric map of the environment while estimating the camera trajectory. As an alternative to feature-based methods, direct methods use the image intensity values instead of extracting features to track the camera trajectory even in featureless environments such as semidense direct VO, called *DSO* [130] and *LDSO* [131], improving the *DSO* by adding loop closure into the optimization pipeline, whereas *LSD-SLAM* [132], *DPPTAM* [133], and *DSM* [134] perform a direct monocular SLAM tracking camera trajectory along with building a semidense model of the environment. Methods have also been presented that combine the advantages of both feature-based and intensity-based methods, such as *SVO* [135] performing high-speed semi-direct *VO*, *CPA-SLAM* [136], and *loosely coupled semidirect SLAM* [137] utilizing image intensity values for optimizing the local structure and image features to optimize the key-frame poses.

Deep Learning models may be used effectively to learn from data to estimate the motion from sequential observations. Hence, their online prediction could be better before initializing the factor-graph optimization problem closer to the correct solution [138,139]. *MagicVO* [140] and *DeepVO* [141] study supervised end-to-end pipelines to learn monocular *VO* from data not requiring complex formulations and calculations for several stages, such as feature extraction and matching, keeping the VO implementation concise and intuitive. There are also some supervised approaches such as *LIFT-SLAM* [142], *RWT-SLAM* [143], and [144,145] that utilize deep neural networks for improved feature/descriptor extraction. Alternatively, unsupervised approaches [146,147,148] exploit the brightness constancy assumption between frames in close temporal proximity to derive a self-supervised photometric loss. The methods have gained momentum, enabling the learning from unlabeled videos and continuously adapting the DL models to newly seen data [149,150]. Nevertheless, monocular visual-only methods suffer from the considerable limitation of being unable to estimate the metric scale directly and accurately track the robot poses in the presence of pure rotational or rapid/acrobatic motion. *RAUM-VO* [151] mitigates the rotational drift by integrating an unsupervised learned pose with the motion estimated with a frame-to-frame epipolar method [152].

**Figure 6:**
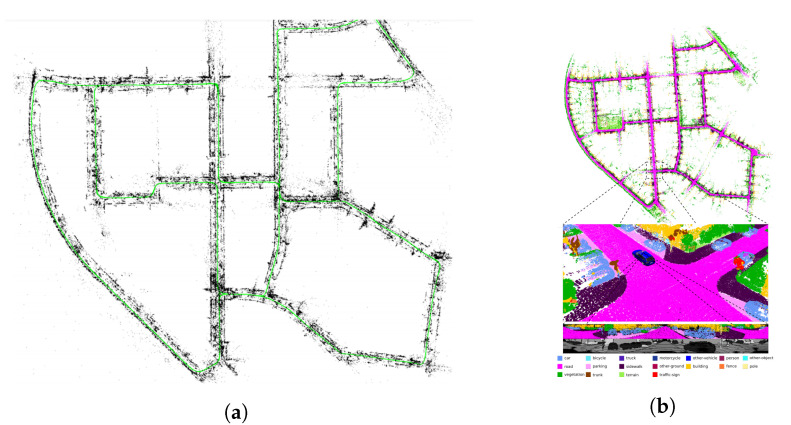
(**a**) Three-dimensional feature map of the environment created using *ORB-SLAM2*. Copyright to [161]. (**b**) The same environment is represented with a 3D semantic map using *SUMA++*, providing richer information to better understand the environment around the robot. Copyright to [182].

To overcome these limitations, cameras are combined with other sensors, for example, synchronizing them with an IMU, giving rise to the research line working on monocular *Visual–Inertial Odometry (VIO)*. Methods such as *OKVIS* [153], *SVO-Multi* [154], *VINS-mono* [155], *SVO+GTSAM* [156], *VI-DSO* [157], *BASALT* [158] are among the most outstanding examples. Delmerico and Scaramuzza [159] benchmarked all the open-source *VIO* algorithms and compared their performance on computationally demanding embedded systems. Furthermore, *VINS-fusion* [160] and *ORB-SLAM2* [161] (see Figure 6a) provide a complete framework capable of fusing either *monocular*, *stereo*, or *RGB-D* cameras with an IMU to improve the overall tracking accuracy of the algorithms. *ORB-SLAM3* [162] presents improvement over *ORB-SLAM2* by performing even multimap SLAM using different visual sensors along with an IMU.

Methods have been presented that perform thermal inertial odometry for performing autonomous missions using robots in visually challenging environments [163,164,165,166]. The authors in *TI-SLAM* [167] not only performed thermal inertial odometry but also provided a complete SLAM back end with thermal descriptors for loop closure detection. Mueggler et al. [168] presented a continuous-time integration of event cameras with IMU measurements, improving by almost a factor of four the accuracy over event-only *EVO* [169]. Ultimate SLAM [170] combines RGB cameras with event cameras along with IMU information to provide a robust SLAM system in high-speed camera motions.

LIDAR odometry and SLAM for creating metric maps have been widely researched in robotics to create metric maps of the environment such as *Cartographer* [171] and *Hector-SLAM* [172], performing a complete SLAM using 2D LIDAR measurements, and *LOAM* [173] and *FLOAM* [174] providing a *parallel LIDAR odometry and mapping* technique to simultaneously compute the LIDAR velocity while creating accurate 3D maps of the environment. SUMA [175] improves the performance over *LOAM* using dense projective ICP over surfel-based maps. To further improve the accuracy, techniques have been presented which combine vision and LIDAR measurement as in *LIDAR-monocular visual odometry (LIMO)* [176] and *LVI-SLAM* [177], combining monocular image tracking with precise depth estimates from LIDAR measurements for motion estimation. Methods such as *LIRO* [178] and *VIRAL-SLAM* [179] couple additional measurements such as *ultrawide band (UWB)* with visual and IMU sensors for robust pose estimation and map building. Other methods such as *HDL-SLAM* [180] and *LIO-SAM* [181] tightly couple IMU, LIDAR, and GPS measurements for globally consistent maps.

While significant progress has been demonstrated using *metric SLAM* techniques, one of the limitations of these methods is the lack of information extracted from the metric representation, such as (1) a lack of *semantic knowledge of the environment*, (2) an *inefficiency in identifying static and moving objects*, and (3) an *inefficiency in distinguishing different object instances*.

**Table 3:** Summary of the significant VSLAM validated over public datasets.

Classification	Sensors	Method	Dataset	Limitations
Metric factor graphs	RGB (Mono)	ORB-SLAM [128]	The New College Dataset [183]	Difficulty in estimating pure rotations Higher error in low-texture environment Suffer from motion bias Scale uncertainty
		DSO [157], LDSO [131]	TUM RGB-D [184], TUM-Mono [185], EuRoC Mav [186], Kitti Odometry [187]	Require constant illumination Require photometric calibration for improved results Scale uncertainty
		LSD-SLAM [132], DPPTAM [133]	TUM RGB-D [184]	
		DSM [134]	EuRoC Mav [186]	
		Semi-Direct VO [137]	EuRoC Mav [186], TUM Mono [185]	Require accurate initialization Scale uncertainty
		MagicVO [140], DeepVO [141]	Kitti Odometry [187]	Higher computational resources Less accurate than the classical VO counterparts
	RGB (mono) RGB (stereo) RGB-D	ORB-SLAM2 [161]	TUM RGB-D [184], EuRoC Mav [186], Kitti Odometry [187]	Lower accuracy in high-speed motions Lower accuracy in low-feature, low-lighting environments Erroneous loop closures in low-feature/similar environments
	RGB (mono) RGB (stereo) RGB-D RGB + IMU	ORB-SLAM3 [162]	TUM VI [188], EuRoC Mav [186]	Lower accuracy in low-feature, low-lighting environments Erroneous loop closures in low-feature/similar environments
	RGB + IMU	VINS-Mono [160]	EuRoC Mav [186]	Requires good initial estimate Lower accuracy in low-feature, low-lighting environments
		SVO-Multi [154]	EuRoC Mav [186], TUM RGB-D [184], ICL-NUIM [189]	Requires robust initialization Flat ground plane assumption causing inaccuracies over nonplanar surfaces
	RGB-D	CPA-SLAM [136]	TUM RGB-D [184], ICL-NUIM [189]	Lower accuracy in case of inaccurate planar detection
Metric–semantic factor graphs	RGB (Mono)	Monocular Object SLAM [190]	TUM RGB-D [184]	Pregenerated object database
		QuadricSLAM [191]	TUM RGB-D [184]	Assumption of scene representation as quadrics Quadric computation computationally expensive Scale uncertainty
		CubeSLAM [192]	TUM RGB-D [184], ICL-NUIM [189]	Lower accuracy in case of higher errors in cuboid detection Scale uncertainty
	RGB (Mono) RGB (Stereo) RGB-D	DynaSLAM [190]	TUM RGB-D [184], Kitti Odometry [187]	Filter out useful dynamic key points No topological relationships between the dynamic–static entities
	RGB-D	VDO-SLAM [193]	Kitti Odometry [187], Oxford Multimotion [194]	No topological relations between the dynamic–static entities in the optimization graph
	RGB (Stereo) + IMU	Kimera [195]	EuRoC Mav [186]	Computationally expensive planar mesh generation No topological constraints between the semantic entities

**Table 4:** Summary of the significant LIDAR-based SLAM validated over public datasets.

Classification	Sensors	Method	Dataset	Limitations
Metric Factor Graphs	LIDAR (2D)	Cartographer [171]	Deutsches Museum [171]	Scan matching can present inaccuracies in cluttered/dynamic environments Loop closure can present inaccuracies in environments with similar structure
	LIDAR (3D)	LOAM [173], FLOAM [174]	Kitti [187]	Inaccuracies in nonstructured environments (without planar/edge features) Inaccuracies in the presence of dynamic objects No explicit appearance-based loop closure
		SUMA [175]	Kitti [187]	Require 3D LIDAR model Errors in loop closures in similar environments Validated mostly on outdoor urban environments
	LIDAR (3D) + RGB (Mono)	LIMO [176]	Kitti [187]	Inaccuracies in low-texture environments Degradation of performance during high-speed motions No loop closure
	LIDAR (3D) + IMU + GPS	HDL-SLAM [180]	Kitti [187]	Scan matching can present inaccuracies in cluttered/dynamic environments Optimization graph contains only robot poses and no environmental landmarks Inaccurate loop closure in similar structured environments
Metric–semantic factor graphs	LIDAR (3D)	LeGO-LOAM [196]	Kitti [187]	High dependence on ground plane Inaccuracies in the presence of features extracted from dynamic objects
		SA-LOAM [171]	Kitti [187], Semantic-Kitti [197], Ford Campus [198]	Limited accuracy in indoor environments Degradation in loop closure in case of noisy semantic detection
		SUMA++ [182]	Kitti [187], Semantic-Kitti [197]	Limited to outdoor urban environments Rely on accurate LIDAR model

#### 4.2.3. Metric–Semantic Factor Graphs

As explained in Section 3, the advancements in *direct situational comprehension* techniques have enabled a higher-level understanding of the environments around the robot, leading to the evolution of *metric–semantic SLAM* overcoming the limitations of traditional *metric SLAM* and providing the robot with the capabilities of human-level reasoning. Several approaches to address these solutions have been explored, which are discussed in the following.

*Object-based metric–semantic SLAM* builds a map of the instances of the different detected object classes on the given input measurements. The pioneer works *SLAM++* [199] and [190] created a graph using camera pose measurements and the objects detected from previously stored database to jointly optimize the camera and the object poses. Following these methods, many object-based *metric–semantic SLAM* techniques were presented, such as [191,192,200,201,202,203,204,205] not requiring a previously stored database and jointly optimizing the camera poses, 3D geometric landmarks, as well as the semantic object landmarks. LIDAR-based metric–semantic SLAM techniques such as LeGO-LOAM [196] extract planar/edges features from semantics such as ground planes to improve the performance over metric SLAM *LOAM* [173]. *SA-LOAM* [206] utilizes semantically segmented 3D LIDAR measurements to generate a semantic graph for robust loop closures. The primary sources of inaccuracies of these techniques are due to an extreme dependence on the existence of objects, as well as (1) the *uncertainty in object detection*, (2) the *partial views of the objects which are still not handled efficiently*, and (3) *no consideration of the topological relationship between the objects*. Moreover, most of the previously presented approaches cannot handle dynamic objects. Research works on filtering dynamic objects from the scene, such as *DynaSLAM* [207], or adding dynamic objects to the graph, such as *VDO-SLAM* [193] and *RDMO-SLAM* [208], reduce the influence of the dynamic objects on the robot pose estimate obtained from the optimized graph. Nevertheless, they cannot handle complex dynamic environments and only generate a sparse map without topological relationships between these dynamic elements.

*SLAM with a metric–semantic map* augments the output metric map given by SLAM algorithms with semantic information provided by *scene understanding* algorithms, as in [209,210,211] or with *SemanticFusion* [212], *Kimera* [195], and *Kimera-Multi* [213]. These methods assume a static environment around the robot; thus, the quality of the *metric–semantic map* of the environment can degrade in the presence of moving objects in the background. Another limitation of these methods is that they do not utilize useful semantic information from the environment to improve the robot’s pose estimation and thus the map quality.

*SLAM with semantics to filter dynamic objects* utilizes the available semantic information of the input images provided by the *scene understanding* module only to filter badly conditioned objects (i.e., moving objects) from pictures given to the SLAM algorithms, as in [214,215,216] for image-based approaches or *SUMA++* [182] (see Figure 6b) for a LIDAR-based approach. Although these methods increase the accuracy of the SLAM system by filtering moving objects, they neglect the rest of the semantic information from the environment to improve the robot’s pose estimation.

### 4.3. Mapping

This section covers the recent works which focus only on the complex high-level representations of the environment. Most of these methods assume the SLAM problem to be solved and focus only on the scene representation. An ideal environmental representation must be efficient concerning the required resources, capable of reasonably estimating regions not directly observed, and flexible enough to perform reasonably well in new environments without any significant adaptations. Table 5 summarizes the main mapping methods described above with their generated map types and limitations.

Occupancy mapping is a method for constructing an environment map in robotics. It involves dividing the environment into a grid of cells, each representing a small portion of the space. The occupancy of a cell represents the likelihood of that cell being occupied by an obstacle or not. Initially, all cells in the map can be considered unknown or unoccupied. As the robot moves and senses the environment, the occupancy of cells is updated based on sensor data. One of the most popular approaches in this category is Octomap [217]. It represents the grid of cells through a hierarchical structure that allows a more efficient query of the occupancy probability in a specific location.

The adoption of Signed-Distance Field (SDF)-based approaches in robotics is well-established to represent the robot’s surroundings [218] and enable a planning of a safe trajectory towards the mission goal [219]. In general, an SDF is a three-dimensional function that maps points of a metric space to the distance to the nearest surface. SDFs can represent distances in any number of dimensions, define complex geometries and shapes with an arbitrary curvature, and are widely used in computer graphics. However, a severe limitation of *SDFs* is that they can only represent watertight surfaces, i.e., surfaces that divide the space between inside and outside [220].

An SDF has two main variations, Euclidean Signed-Distance Field (ESDF) and Truncated Signed-Distance Field (TSDF), which usually apply to a discretized space made of voxels. On the one hand, an ESDF gives the distance to the closest obstacle for free voxels and the opposite for occupied ones. They have been used for mapping in *FIESTA* [221], where the authors exploited the property of direct modeling free space for collision checking and the gradient information for planning [222], while dramatically improving their efficiency. On the other hand, a TSDF relies on the projective distance, which is the length measured along the sensor ray from the camera to the observed surface. The distances are calculated only within a specific radius around the surface boundary, known as the truncation radius [223]. This helps improve computational efficiency and reduce storage requirements while accurately reconstructing the observed scene. TSDFs have been demonstrated in multiple works such as *Voxgraph* [224], *Freetures* [225], *Voxblox++* [226], or the more recent *Voxblox-Field* [227]. They can create and maintain globally consistent volumetric maps that are lightweight enough to run on computationally constrained platforms and demonstrate that the resulting representation can navigate unknown environments. A panoptic segmentation was rated with TSDFs by Narita et al. [228] for labeling each voxel semantically while differentiating between *stuff*, e.g., the background wall and floor, from *things*, e.g., movable objects. Furthermore, Schmid et al. [229] leveraged pixelwise semantics to maintain temporal consistency and detect changes in the map caused by movable objects, hence surpassing the limitations of a static environment assumption.

*Implicit neural representations (INR)* (sometimes also referred to as coordinate-based representations) are a novel way to parameterize signals of all kinds, even environments parameterized as 3D points clouds, voxels, or meshes. With this in mind, *scene representation networks (SRNs)* [230] have been proposed as a continuous scene representation that encodes both geometry and appearance and can be trained without any 3D supervision. It has been shown that *SRNs* generalize well across scenes, can learn geometry and appearance priors, and are helpful for novel view synthesis, few-shot reconstruction, joint shape, and appearance interpolation in the unsupervised discovery of nonrigid models. In [231], a new approach was presented, capable of modeling signals with fine details and accurately capturing their spatial and temporal derivatives. Based on periodic activation functions, that approach demonstrated that the resulting neural networks referred to as *sinusoidal representation networks (SIRENs)* were well suited for representing complex signals, including 3D scenes.

*Neural radiance fields (NeRF)* [232] exploit the framework of *INR*s to render realistic 3D scenes by a differential process that takes as input a ray direction and predicts the color and density of the scene’s structure along that ray. Sucar et al. [233] pioneered the first application of *NeRF* to SLAM for representing the knowledge of the 3D structure inside the weights of a deep NN. For their promising results, that research prospect attracted numerous following works that continuously improved the fidelity of the reconstructions and the possibility of updating the knowledge of the scene while maintaining previously stored information [234,235,236,237,238].

Differently from the previous dense environment representation methods, which are helpful for autonomous navigation, sparser scene representations also exist, such as *point clouds* and *surfel maps*, which are more commonly used for more straightforward tasks such as localization. Remarkably, a *surfel*, i.e., a surface element, is defined by its position in 3D space, the surface normal, and other attributes such as color and texture. Their use has been extensively explored in recent LIDAR-based SLAM to efficiently represent a 3D map that can be performed as a consequence of optimization following revisited places, i.e., loop closure [239,240].

Three-dimensional *scene graphs* have also been researched to represent a scene, such as in [241,242,243], which build a model of the environment, including not only metric and semantic information but also essential topological relationships between the objects of the environment. They can construct an environmental graph spanning an entire building, including the semantics of objects (class, material, and shape), rooms, and the topological relationships between these entities. However, these methods are executed offline and require an available 3D mesh of the building with the registered RGB images to generate the *3D scene graphs*. Consequently, they can only work in static environments.

**Figure 7:**
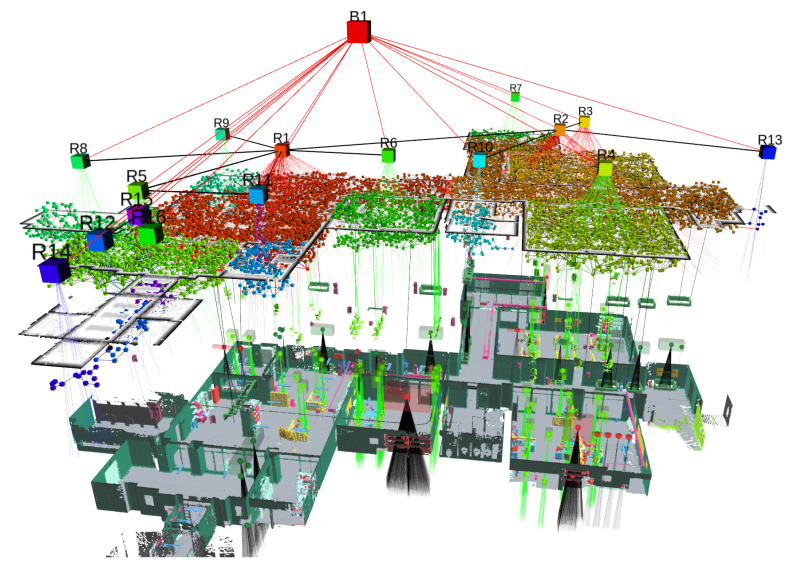
A Dynamic Scene Graph generated by [245] with a multilayer abstraction of the environment. Copyright to [245].

Dynamic Scene Graphs (DSGs) (see Figure 7) are an extension of the aforementioned *scene graphs* to include dynamic elements (e.g., humans) of the environment in an actionable representation of the scene that captures geometry and semantics [244]. Rosinol et al. [245] presented the first method to build a DSG automatically using the input of a VIO [195]. Furthermore, it allowed the tracking of the pose of humans and optimized the mesh based on the deformation of the space induced by detected loop closures. Although these results were promising, their main drawback was that the DSG was built offline, and the VIO first created a 3D-mesh-based semantic map fed to the dynamic scene generator. Consequently, the SLAM did not use these topological relationships to improve the accuracy of the spatial reconstruction of the robot trajectory. Moreover, except for humans, the remaining topological relationships were considered purely static, e.g., chairs or other furniture were fixed to the first detection location.

**Table 5:** Summary of the significant types of mapping algorithms and their limitations.

Mapping Type	Sensors	Methods	Limitations
Occupancy maps	RGB-D 2D LIDAR 3D LIDAR	Octomap [217]	Cannot represent the exact shape and orientation of objects Increased complexity in map querying with increase map size No semantics
ESDF and TSDF	RGB-D 3D LIDAR	Voxblox [223]	No Semantics ESDF map updates can present errors during loop closures
		Voxgraph [224]	No semantics Degradation of the esdf map quality in the presence of noisy odometry estimates
		Voxblox++ [226]	Degradation of the esdf map quality in the presence of noisy odometry estimates Computationally expensive semantic detection Degradation in map quality with noise in the semantic detection
NeRF	RGB-D	iMap [233], Urban Radiance Fields [246], Mega-NeRF [247]	No semantics (but potentially learnable) Computationally expensive Needs to handle catastrophic forgetting while integrating new knowledge
Surfel maps	RGB-D 3D LIDAR	ElasticFusion [239], SurfelMeshing [248], Other [240]	Sparse representation Cannot represent continuous surfaces Less useful for path planning and obstacle avoidance
3D Scene Graphs	RGB-D	3D DSG [241], Hydra [249]	Validated mainly in indoor scenarios Handle few dynamic objects in the scene, such as humans

More recently, Hydra [249] has implemented the scene graph construction into a real-time capable system relying on a highly parallelized architecture. Moreover, it can optimize an embedded deformation graph online, after a loop closure detection. Remarkably, the information in the graph allows the creation of descriptors based on histograms of objects and visited places that can be matched robustly with previously seen locations.

Therefore, DSGs, although in their infancy stage, are shown to be a practical decision-making tool that enables robots to perform autonomous tasks. For example, Ravichandran et al. [250] demonstrated how they could be used for learning a trajectory policy by turning a DSG into a graph observation that served as input to a Graph Neural Network (GNN). A DSG may also be used for planning challenging robotic tasks, as proposed in the Taskography benchmark [251].

Lastly, one of the main features of a DSG is the possibility to perform queries and predictions of the future evolution of the scene based on dynamic models linked with the agents or physical elements [244]. In addition, an even more intriguing property is their application to scene change detection or to the newly formalized *semantic scene variability estimation* task, which sets as a goal the prediction of long-term variation in location, semantic attributes, and topology of the scene objects [252]. This property has only been explored by scratching the surface of its potential application. Still, it already grasps our vision of a comprehension layer that produces the knowledge required by the projection and prediction of future states.

## 5. Situational Projection

In robotics, the projection of the situation is essential for reasoning and the execution of a planned mission [253]. The comprehended information can be projected in the future to predict the future state of the robot by using a dynamic model [101] as well as the dynamic entities in the environment. In order to predict the future state of a robot, the projection component requires more effort in producing models that can forecast the dynamic agents’ behavior and how the scene is affected by changes that shift its appearance over time. Remarkably, numerous research areas address specific forecast models, the interactions between agents, and the surrounding environment’s evolution. Below, we give an overview of the most prominent ones that we deem more related to the robotic SA concept.

### Behavior Intention Prediction

Behavior intention prediction (BIP) focuses on developing methods and techniques to enable autonomous agents, such as robots, to predict the intentions and future behaviors of humans and other agents they interact with. This research is essential for effective communication, collaboration, and decision-making. BIP typically involves integrating information from multiple sources, such as visual cues, speech, and contextual information, e.g., coming from the comprehension layer. This research has numerous applications, including human–robot collaboration, autonomous driving, and healthcare. Especially regarding the Autonomous Vehicle (AV) application, this topic has gained importance among researchers and is widely studied due to safety concerns. However, we argue that the outcome of its investigation may apply to other tasks implying an interaction among a multitude of agents.

To define the AV BIP task, we refer to the recent survey [254] that distinguishes various research topics related to understanding the driving scene under a unified taxonomy. The whole problem is then defined by analyzing on a timeline the events happening on the road scenario and the decision-making factors that lead to specific outcomes.

Scene contextual factors, such as traffic rules, uncertainties, and interpretation of goals, are crucial for inferring the interaction among road actors [255] and the safety of the current driving policy [256]. Specifically, interaction may be due to social behavior or physical events such as obstacles or dynamic clues, e.g., traffic lights, that influence the decision of the driver [257]. Multimodal perception is exploited to infer whether pedestrians are about to cross [258] or vehicles to change lanes [259]. Mostly, recent solutions rely on DL models such as CNNs [260,261], Recurrent Neural Networks (RNNs) [257,262], GNNs [263], or on the transformer attention mechanism, which can estimate the crossing intention using only pedestrian bounding boxes as input features [264]. Otherwise, causality relations are studied by explainable AI models to make risk assessment more intelligible [265]. Lastly, simulation tools of road traffic and car driving, such as CARLA [266], can be used as forecasting models provided that mechanisms to adapt synthetically generated data to the reality are put in place [267].

BIP then requires fulfilling the task of predicting the trajectory of the agents. For an AV, the input to the estimation is represented by the historical sequence of coordinates of all traffic participants and possibly other contextual information, e.g., velocity. The task is then to generate a plausible progression of the future position of other pedestrian vehicles. Methods for predicting human motion have been exhaustively surveyed by Rudenko et al. [268], and regarding vehicles, by Huang et al. [269], who classified the approaches into four main categories: physics-based, machine learning, deep learning, and reinforcement learning. Moreover, the authors determined the various contextual factors that may constitute additional inputs for the algorithms similar to those previously described. Finally, they acknowledged that complex deep learning architectures were the de facto solution for real-world implementation for their performance.

Additionally, DL allows for multimodal outputs, i.e., the generation of a diverse trajectory with an associated probability, and for multitask learning, i.e., simultaneously producing a likelihood of a specific behavior. Behavior prediction is, in fact, a separate task, more concerned with assigning to the road participants an intention of performing a particular action. In the recent literature, we can find reviews of approaches specific to understanding the behaviors of vehicles [270] and pedestrians [271]. Behavior prediction is also related to forecasting the occurrence of accidents. This capability is a highly demanded skill in many industrial scenarios.

## 6. Discussion

In the previously presented sections, we thoroughly reviewed the state-of-the-art techniques offered by the scientific community to improve the overall intelligence of autonomous robotic systems. Importing the knowledge from psychology to robotics, we showed that a situational awareness perspective in robotics can efficiently classify these presented state-of-the-art techniques in an organized and multilayered manner. Consequently, we addressed the research questions posed at the beginning:

Through the literature review, we found a gap between the presented approaches to provide a unified and complete Situational Awareness for the robots to understand and reason about the environment in order to perform a mission autonomously and closely to how human beings would. To this end, we proposed an ideal model of the robotic SA system, which, per our mentioned conventions, would be divided into three subsections. The **perception layer** should consist of a multimodal sensor suite for accurate environmental perception. The **comprehension layer** may bear methods from *direct situational comprehension* and *accumulated situational comprehension* to improve the robot’s ego-awareness of its state, such as its pose, but also model the external factors with which it interacts, e.g., objects and the environment 3D structure, in the form a metric–semantic topological scene graphs. Finally, the **projection layer**, which still has few connections with the underlying perception and comprehension and is usually treated on a standalone basis, would add forecasting models to predict the future state of the robot as well as the dynamic environmental elements.

The progress in AI and DL has been pivotal in enhancing the robot’s comprehension of a situation, as depicted by previous research. Despite significant progress in mobile robots’ *direct situational comprehension*, a versatile and standardized sensor suite must be developed to handle environmental challenges, such as meteorological changes. Additionally, integrating these algorithms efficiently into scene modeling frameworks remains a challenge.

The scene graph presented in the related works is a widely adapted term in computer vision [272] to describe the relationship between objects in a scene with a structured representation between entities and predicates usually built from visual information. However, we saw the current scene graphs used in mobile robotics were insufficient to address complex autonomous tasks, such as multimodal open-set queryable maps for navigation [273,274]. Therefore, starting from the scene graph concept, we introduced the *S-Graph* as a knowledge graph that emphasized the ability to store the entire representation of the situation, comprising the currently perceived aspects of the scene, their comprehension, the integration with previous records or possibly also external sources from a standardized ontology [275], and the prediction of the future by the projection of the entities through their models.

**Figure 8:**
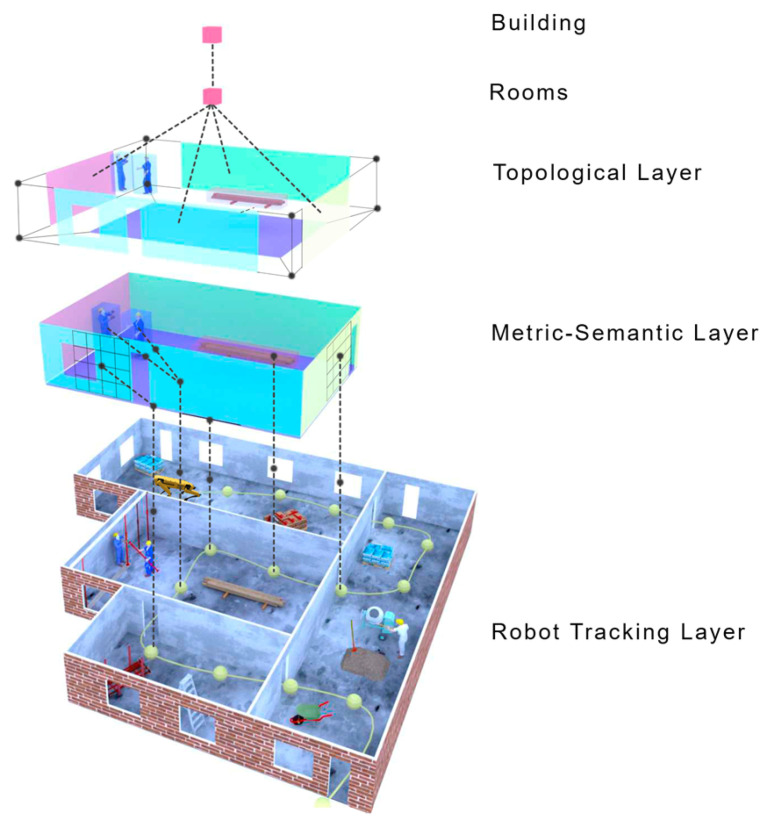
Proposed *S-Graph*. The graph is divided into three sublayers: the tracking layer, which tracks the sensor measurements as creates a local key-frame map containing its respective sensor measurements; the metric–semantic layer creates a metric–semantic map using the local key frames; the topological layer consists of the topological connections between the elements in a given area and the rooms that connect planar features.

Hence, we set the *S-Graph* (see Figure 8) as a future target of the evolution of current scene graphs, which adds a hierarchy of conceptual layers that contribute to including prior knowledge of the situation while maintaining their formulation. Furthermore, the current implementation of the *S-Graph* [276,277], which is still in its initial stage, stresses the practical characteristic of using the created entity relationships, e.g., topological aspect, to obtain an optimized answer on the state of an autonomous agent, e.g., the robot’s pose.

We believe this approach can accelerate progress and improve the autonomy of mobile robots.

## 7. Conclusions

In this paper, we argued that Situational Awareness is an essential capability of humans that has been studied in several different fields but has barely been considered in robotics. Instead, robotic research has focused on ideas in a diversified manner, such as sensing, perception, localization, and mapping. Thus, as a direct line of future work, we proposed a three-layered Situational Awareness framework composed of perception, comprehension, and projection. To this end, we provided a thorough literature review of the state-of-the-art techniques for improving robotic intelligence. Then, we reorganized them in a more structured, layered perception, comprehension, and projection format.

Finally, we conclude by providing appropriate answers to the earlier research question.

- *What has been achieved so far, and what challenges remain?*
  Given the advancements in AI and DL, we notice an improved comprehension layer by evaluating state-of-the-art algorithms. Comparing the initial approaches relying on heuristics and heavily engineered processing, current algorithms can solve complex tasks requiring generalization and adaptation in dynamic environments. Nevertheless, the algorithms follow a compartmentalized approach impeding a unified SA for mobile robots. Remarkably, forecasting the future situation is also in its infancy and relies on perfect data from the perception and comprehension layers to demonstrate meaningful results.
- *What could the future direction of Situational Awareness be?*
  We argue that after analyses of these algorithms, a situational awareness perspective can steer robots towards a faster achievement of their tasks, by comprising multimodal hierarchical *S-Graphs* generating a metric–semantic topological map of its environment as well as improving the robot’s pose uncertainty in it. We foresee the *S-Graph* will be characterized by a tighter coupling of situational projection, perception, and comprehension, to complete the transition from static world assumptions to natural dynamic environments.

## Figures and Tables

**Table 1:** Different types of sensors utilized onboard mobile robots for situational perception.

Classification	Sensor	Measurement	Mobile Robotic Platforms	Limitations	Examples
Proprioceptive	IMU	Velocities Accelerations Yaw angle (with a magnetometer)	Indoor/outdoor robots	Bias Gaussian noise Drifts rapidly	MPU-6050
	GPS	Absolute position	Outdoor robots	Unreliable measurements in cluttered environments.	u-blox NEO-M8N
	Barometer	Altitude from atmospheric pressure	Indoor/outdoor aerial robots	Bias Gaussian noise	Bosch BMP280
	Robot encoders	Relative position Velocity	Indoor/outdoor ground robots	Slippage Error accumulation	US Digital E4P
	RF Receiver	Absolute position	Indoor/outdoor robots	Prone to interference Limited range	DecaWave DWM1000
Exteroceptive	RGB camera	Visible light	Indoor/outdoor robots	Motion blur Degradation in poor light conditions	IDS uEye LE
	RGB-D Camera	Visible light Depth from IR structured light	Indoor/outdoor robots	Limited and noisy range Errors in reflective/transparent surfaces	Intel Realsense D435
	IR Camera	Infrared radiation	Indoor/outdoor robots	Limited information Prone to atmospheric interference Infrared cannot pass through glass or water	FLIR Lepton
	Event camera	Brightness log-intensity changes	Indoor/outdoor robots	Requires motion of camera or objects Absolute brightness not measured directly Not easy to purchase	DAVIS 346 or SONY IMX636ES
	LIDAR	Metric distances and angle of scene points	Indoor/outdoor robots	Prone to atmospheric interference Degradation in reflective and transparent surfaces	Velodyne VLP-16
	MmWave FMCW RADAR	Metric distances and angle of scene points Objects’ speed	Indoor/Outdoor Robots	Limited range and field of view Possible low angular and distance resolution Multipath propagation effect and ghost targets	AWR6843AOP

**Table 2:** Summary of the algorithms for the direct situational comprehension SA module. DL refers to methods leveraging deep learning.

Modality	Sensor	Method	DL	Limitations	References
Monomodal	RGB	Feature detection	✗	Sensitive to illumination changes Higher false positives Lower robustness in the presence of occlusions	[25,26,27,28,29,30,31,32,33,34]
		Object detection	✓	Higher computation cost Larger training dataset Sensitive to occlusions No instance segmentation	[35]
		Semantic segmentation	✓	Higher computation cost Larger training dataset No instance segmentation	[36,37,38,39,40,41,42,43,44]
		Panoptic segmentation	✓	Higher computation cost Larger training dataset Slower inference time	[45,46]
		2D Scene graphs	✓	Limited to 2D spatial information Limited temporal information	[47,48,49]
	Thermal	Object detection	✗	Limited applicability Higher false positives	[50]
		Object detection	✓	Limited applicability Limited datasets	[51,52]
	Event	Object detection	✓	Trained over limited data Limited validation in the presence of occlusions	[53,54]
		Semantic segmentation	✓	Trained over limited data Limited to only a few semantic objects	[54]
	LIDAR	Object detection	✗	Detection of fewer semantic entities Lower robustness in the presence of outliers and occlusions	[55,56,57]
		Semantic segmentation	✓	Limited to fewer semantic entities Higher computational cost Lower accuracy in indoor environments	[58,59,60,61,62,63,64,65,66]
Multimodal	RGB + Depth	Object detection	✗	Higher false positives and negatives Limited range	[67,68]
		Object detection	✓	Limited range and limited to low-range applications High computation cost Lower inference time Lack of generalizability over nontrained semantic entities	[69,70,71,72,73]
	RGB + Thermal	Semantic Segmentation	✓	Limited real world testing Mostly limited to outdoor environments Lower object detection accuracy	[74,75,76,77]
	RGB + Event	Semantic segmentation	✓	Tested only on outdoor datasets	[78]
	RGB + LIDAR	Object detection	✓	Limited accuracy in indoor environments No temporal history of detected objects for efficient tracking	[79,80,81,82,83]
